# Spontaneous Auto-Amputation of the Breast in a Patient With Advanced Malignant Fungating Wound and Distant Metastases Involving the Lungs and Left Upper Extremity: A Case Report

**DOI:** 10.7759/cureus.106925

**Published:** 2026-04-12

**Authors:** Emilia Piaszczynska, Oktawia Pylak-Piwko

**Affiliations:** 1 Faculty of Medicine, Medical University of Lublin, Lublin, POL; 2 Unit of Palliative Medicine, Faculty of Health, Medical University of Lublin, Lublin, POL

**Keywords:** auto-amputation, breast cancer, fungating malignant wound, neuropathic pain, palliative care

## Abstract

Malignant fungating wounds in advanced breast cancer cause significant morbidity and psychological distress. Spontaneous auto-amputation of the breast is a rare clinical phenomenon, conventionally linked to neglected tumors rather than actively treated disease. We present a case of spontaneous auto-amputation in an extensively pretreated 60-year-old female with advanced human epidermal growth factor receptor 2-positive (HER2+) breast cancer. She presented to the palliative care unit with refractory left upper extremity pain, severe tactile and thermal allodynia due to malignant brachial plexopathy, and extensive malignant lymphedema complicated by deep vein thrombosis. During her clinical course, despite prior multi-line systemic therapy, rapid local tumor proliferation outpaced neovascularization, leading to critical ischemia, extensive necrosis, and the culminating spontaneous auto-amputation of her left breast.

Symptom control required an escalation of pharmacological therapy beyond standard opioid protocols due to inadequate efficacy. To manage the refractory neuropathic pain, the regimen was supplemented with advanced intravenous adjuvant analgesia, bypassing the traditional three-step World Health Organization analgesic ladder. This utilized targeted infusions of lidocaine, magnesium sulfate, and dexmedetomidine. Ultimately, the patient's progressive condition necessitated the implementation of palliative analgosedation to manage refractory distress and maintain dignity. This case highlights that effective advanced management requires dynamic, multimodal palliative strategies, prioritizing the early recognition of complex neuropathic mechanisms and the utilization of advanced intravenous interventions.

## Introduction

Locally advanced breast cancer can manifest with severe cutaneous involvement, leading to the formation of malignant fungating wounds, which impair the patient's physical and psychosocial equilibrium. Spontaneous auto-amputation of the breast is a rare manifestation of such rapidly progressive disease courses, occurring when the unrestrained proliferation of neoplastic cells overwhelms local angiogenesis, leading to ischemia, necrosis, and eventual physical separation of the tissue [1]. While this clinical phenomenon has been documented in modern case reports [2], historically, the majority of cases were associated with invasive ductal carcinoma in medically underserved populations, accompanied by medical delays [3]. To our knowledge, this is among the first reported cases of spontaneous auto-amputation occurring in a patient during active systemic therapy.

The management of modern auto-amputation cases is further complicated by severe neuropathic cancer pain, a condition characterized by high prevalence, hyperalgesia, and a complex pathophysiology involving direct tumor invasion of peripheral nerves, central wind-up phenomena, and treatment-related neural toxicity [4]. Such symptoms affect the physical, emotional, and psychological domains of quality of life, engendering a state of total pain in patients already suffering from complications like extensive upper limb lymphedema and social isolation [5]. Modern locoregional therapies for axillary disease aim to de-escalate morbidity, yet treatment-related sequelae remain a challenge [6].

Achieving optimal pain management in the modern oncology era requires a comprehensive framework that systematically addresses the biological barriers to adequate symptom control and utilizes specialized palliative strategies [7]. Current clinical practice guidelines, such as those propagated by the European Society for Medical Oncology (ESMO), emphasize the necessity of comprehensive pain assessment and the escalation to multimodal analgesia when standard protocols fail to improve outcomes [8]. Understanding the biological dynamics of high-grade malignancies and the complex pathophysiology of neuropathic pain they induce is essential for tailoring these palliative interventions [9].

Furthermore, while microsurgical approaches like lymphaticovenous anastomosis exist for lymphedema in breast cancer survivors, their role in advanced settings with systemic thrombosis is contraindicated, necessitating a shift toward conservative and comfort-oriented palliative paradigms [10].

## Case presentation

A 60-year-old female patient with a history of advanced left-sided breast cancer (cT4N3M1) was admitted to the inpatient palliative care department. The primary tumor was characterized by estrogen receptor (ER) negativity, progesterone receptor (PgR) negativity, and human epidermal growth factor receptor 2 (HER2) positivity, with a high Ki-67 proliferation index of 90%. Her oncological history was significant for aggressive disease with distant metastases to the lungs, managed initially according to the principles of the traditional World Health Organization (WHO) analgesic ladder. Prior systemic treatment was extensive, beginning with 12 cycles of pertuzumab, trastuzumab, and docetaxel, followed by maintenance therapy with pertuzumab and trastuzumab. Following disease progression, she received trastuzumab deruxtecan and bisphosphonates. During the course of palliative management, an oral capecitabine regimen (500 mg + 500 mg + 150 mg daily) was also administered. Notably, the patient had no history of local radiotherapy, excluding radiation-induced vascular damage as a primary etiology for subsequent tissue necrosis.

Upon initial admission, her primary complaints were refractory pain in the left upper extremity coupled with extensive, indurated edema. Clinical presentation and vascular imaging were consistent with deep vein thrombosis occluding the left brachial and radial veins, for which therapeutic enoxaparin was initiated. This vascular occlusion further compounded the pre-existing malignant lymphedema caused by axillary nodal involvement. Managing the hydrostatic pressure gradient was challenging, as standard pneumatic compression devices were contraindicated not only due to the risk of pulmonary embolism in the presence of venous thrombosis but also because mechanical compression would trigger severe neuropathic pain. To facilitate passive lymphatic drainage and maximize patient comfort, the affected limb was elevated using soft supports, as standard orthopedic devices were poorly tolerated in this hospice setting.

**Figure 1:**
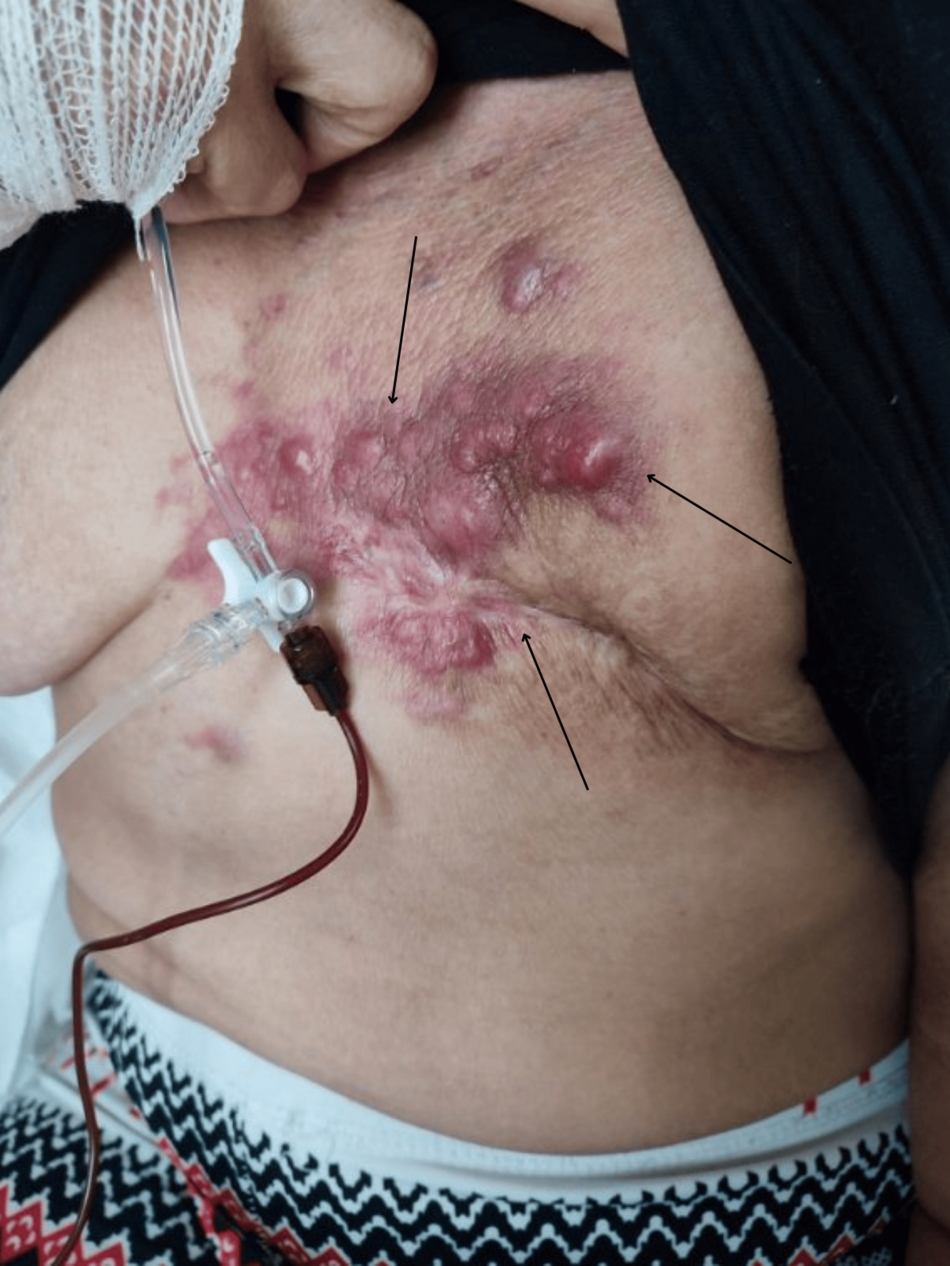
Clinical status of the anterior chest wall six weeks prior to the severe clinical deterioration. The photograph demonstrates signs of previously initiated auto-amputation of the left breast (black arrows), alongside early cutaneous erythematous nodules and ulcerations.

During her prolonged stay at the palliative care department, despite the administration of active systemic therapies, the disease exhibited rapid local progression. Written informed consent was obtained from the patient for the publication of all clinical images. To illustrate the disease trajectory, clinical photographs taken approximately six weeks prior to the severe clinical deterioration were evaluated. At that time, the anterior chest wall already exhibited signs of previously initiated auto-amputation of the left breast, accompanied by early cutaneous involvement with erythematous nodules and ulcerations on the remaining tissue and sternum (Figure 1).

**Figure 2:**
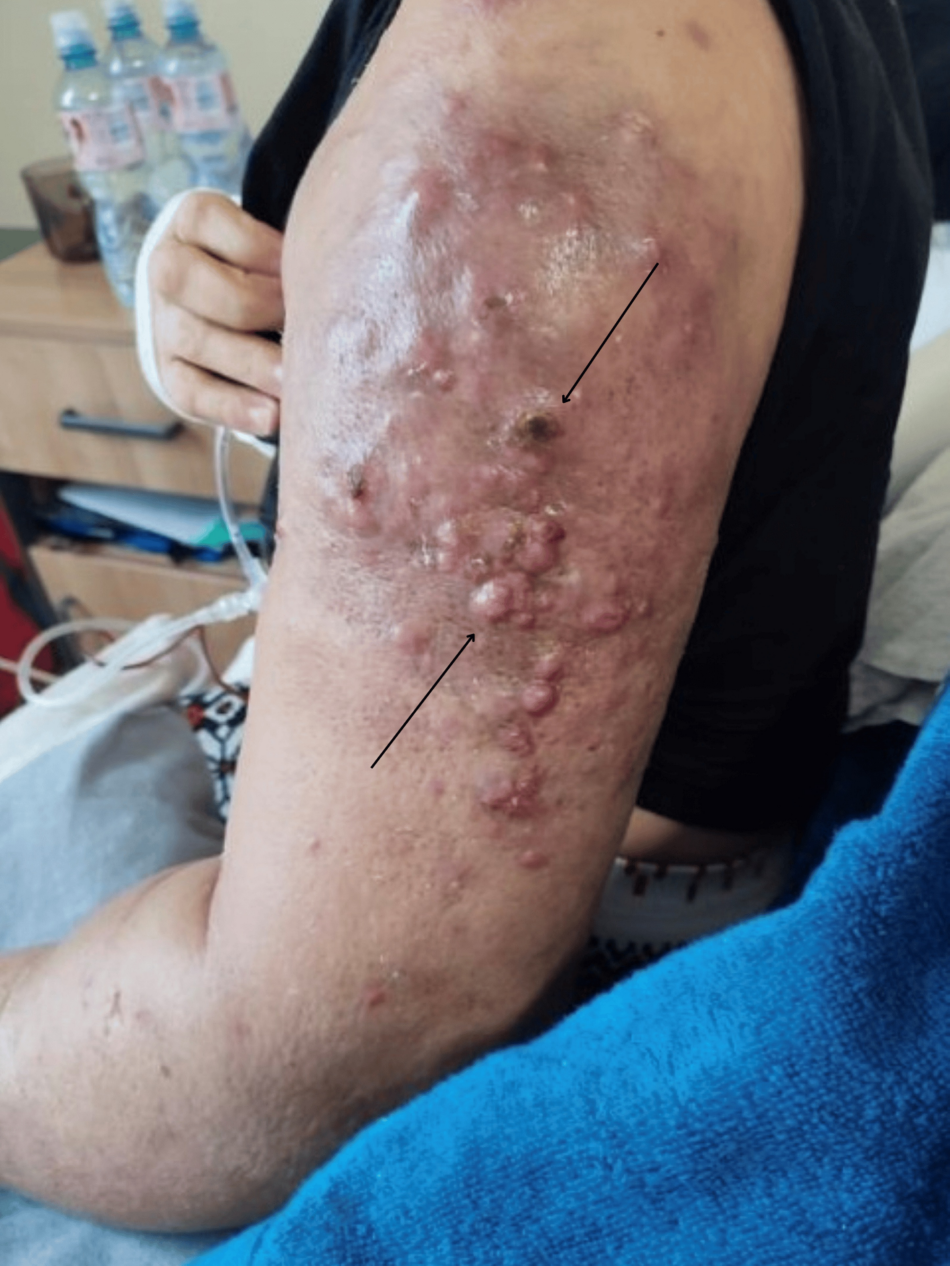
Clinical status of the left upper extremity six weeks prior to the severe clinical deterioration. The limb exhibits mild edema, mostly restricted to the upper arm, with scattered early metastatic nodules and skin discoloration (black arrows highlight representative early lesions).

Concurrently, the left upper extremity showed mild edema restricted mostly to the proximal arm, accompanied by scattered early metastatic nodules and discoloration (Figure 2).

**Figure 3:**
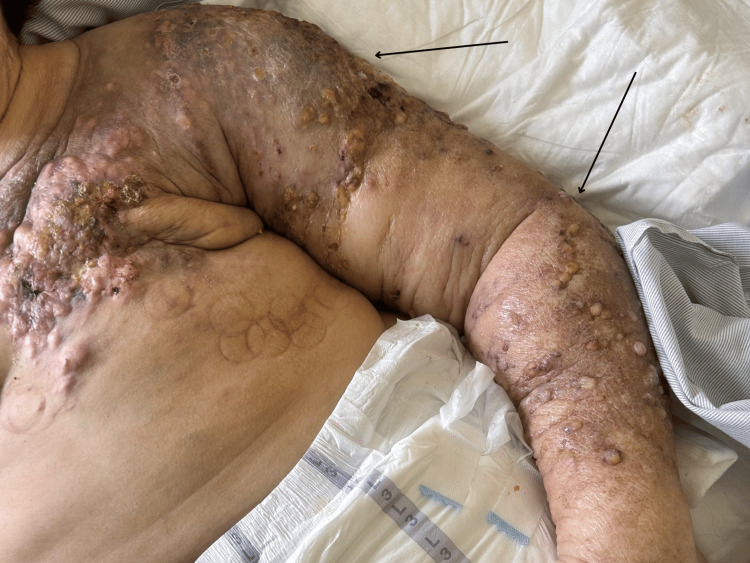
Current stage of severe clinical deterioration on the chest and left upper extremity. The photograph illustrates extensive, confluent metastatic nodules with yellowish necrotic exudate (black arrows), and profound, indurated malignant lymphedema of the left arm.

Over the subsequent six weeks, the disease exhibited explosive local progression. The skin rapidly erupted into massive, confluent, exudative satellite lesions with yellowish necrotic discharge, demonstrating a “nodule-on-top-of-nodule” growth pattern. The left upper extremity developed profound indurated malignant lymphedema (Figure 3).

**Figure 4:**
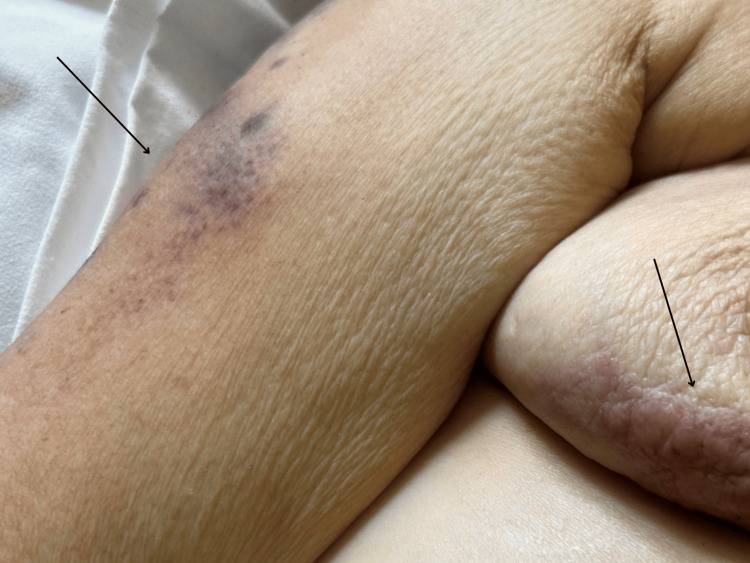
Current stage of severe clinical deterioration on the right upper extremity and contralateral breast. The right arm remains entirely free of lymphedema, presenting with early-stage ulcerating nodules. Additionally, new metastatic cutaneous lesions are now visible on the right breast (black arrows highlight representative nodules on both the arm and the breast). This stark contrast visualizes the severe mechanical and vascular compromise isolated to the left axillary region.

Cutaneous metastatic satellite lesions also developed on the contralateral side, including the right breast. In stark contrast to the left limb, the right upper extremity remained entirely free of lymphedema, presenting only with early-stage ulcerating nodules alongside the progressing lesions on the right breast (Figure 4).

As the disease progressed, the underlying ischemic and necrotic process - driven by tumor growth compromising the vascular supply - culminated in the partial, spontaneous auto-amputation of her left breast. There were no clinical signs of systemic sepsis or acute local infection contributing to the necrotic process. This event left an extensive, complex malignant fungating wound across the anterior chest wall, which required intensive topical management to control local symptoms. Palliative local wound care prioritized the management of malodor through the topical application of metronidazole gel and the absorption of exudate using alginate and silver-impregnated foam dressings, taking into careful account the multidimensional factors that impact patients' experiences and the preservation of body image.

Simultaneously, the patient developed signs of malignant brachial plexopathy, presenting with tactile and thermal allodynia, where even minor exposure to ambient cold elicited severe neuropathic pain. Upon her initial admission to the palliative care unit, her pain intensity was measured at 5/10 on the Numeric Rating Scale (NRS). It is noteworthy that this baseline score was recorded while the patient was already receiving chronic, albeit unspecified, opioid therapy prior to her palliative care admission. This pain was transiently relieved by the application of warm compresses. Baseline inpatient analgesia consisted of transdermal fentanyl at 50 µg/h, extended-release oral oxycodone (40 mg in the morning, 20 mg after lunch, and 20 mg in the evening), and intravenous dexamethasone (40 mg twice daily). This was supplemented with subcutaneous morphine boluses, escalated up to 120 mg/day. Additionally, oral pregabalin was introduced and titrated to the maximum tolerated dose. While the neuropathic pain remained refractory despite these measures, the combination of morphine and dexamethasone effectively alleviated the progressively worsening dyspnea.

To break the neuropathic central sensitization cycle, the regimen was systematically supplemented with advanced intravenous adjuvant analgesia. This included a continuous intravenous infusion of lidocaine at a calculated dose of 200 mg daily (administered with strict electrocardiogram (ECG) and clinical toxicity monitoring), combined with intravenous magnesium sulfate at 2 g to provide N-methyl-D-aspartate (NMDA) receptor antagonism. This combined blockade significantly mitigated the cold-induced allodynia, reducing the NRS pain score from 5/10 to 3/10 within three days of intervention and achieving sustained analgesic efficacy at 1-2/10 over the subsequent 20 days.

Despite comprehensive local care and adequate analgesia, the systemic burden of the metastatic disease continued to impact the patient's status. To manage disease-related restlessness and refractory distress during the final stages of her disease trajectory, palliative analgosedation utilizing a continuous infusion of dexmedetomidine (dosed at a range of 0.2 to 0.7 mcg/kg/hr) was implemented. This intervention provided light, awake sedation, allowing for comfort without inducing respiratory depression. The initiation of palliative sedation was strictly aligned with institutional and national ethical guidelines, and informed consent was formally obtained by the attending physician prior to the intervention.

## Discussion

Spontaneous auto-amputation of the breast is a rare oncological complication. Historical literature primarily links this phenomenon to slow-growing tumors and medical delay [1-3]. In this case, auto-amputation occurred despite targeted multi-line systemic therapy. Figures 1-4 document the disease trajectory. Following the initial tissue separation, rapid local progression into massive exudative lesions and severe lymphedema occurred over a six-week period. This presentation demonstrates the biological behavior of specific HER2+ phenotypes, where tumor cellular proliferation outpaces the vascular supply, resulting in ischemia and necrosis [11,12]. Concurrently, extensive malignant lymphedema and acute upper extremity deep vein thrombosis created a severe hydrostatic pressure gradient. Physical compression therapies were contraindicated due to the risk of pulmonary embolism and the potential exacerbation of neuropathic pain [4-6].

Consequently, pain management was the primary clinical challenge [7-10]. The patient exhibited signs of malignant brachial plexopathy secondary to mechanical compression and neoplastic nerve invasion [13]. Standard opioid protocols were insufficient for managing central sensitization. The integration of intravenous lidocaine and magnesium sulfate provided a targeted biochemical blockade of hyperexcitable nerve fibers. Intravenous lidocaine is utilized in refractory neuropathic cancer pain to silence ectopic nociceptive firing from voltage-gated sodium channels. Magnesium sulfate acts as a physiological calcium channel blocker, antagonizing the NMDA receptor and disrupting opioid resistance and central wind-up phenomena [14].

Although the neuropathic pain was opioid-refractory, the administration of intravenous dexamethasone and systemic morphine effectively managed the progressive dyspnea. Additionally, the introduction of dexmedetomidine facilitated palliative analgosedation. As an alpha-2 adrenergic agonist, it provided awake sedation and synergized with the analgesic regimen, maximizing the opioid-sparing effect without inducing respiratory depression. Concurrently, non-curative wound management focused on odor control via topical antimicrobials and exudate absorption. This local wound care maintained patient comfort and quality of life in the setting of advanced disease [15].

Limitations

While this case provides valuable clinical insights, several limitations must be acknowledged. Primarily, as a single case report, the findings have limited generalizability to broader patient populations. Although NRS scores were retrospectively evaluated, the lack of more comprehensive, objective neuropathic pain scoring systems (such as the DN4 or LANSS questionnaires) at baseline limits the qualitative assessment of the pain phenotype. Furthermore, due to the advanced stage of the disease and the palliative setting, there is a lack of long-term outcome data regarding the sustainability of the described analgesic interventions.

Summary

To encapsulate the core clinical takeaways from this specific presentation, the key learning points are summarized as follows: (i) auto-amputation can occur even in treated aggressive cancers; (ii) neuropathic pain may be opioid-refractory; (iii) lidocaine + magnesium can break central sensitization; (iv) dexmedetomidine enables awake sedation; (v) multimodal palliative care is essential.

## Conclusions

Spontaneous auto-amputation of the breast is a rare clinical manifestation of high-proliferative oncological disease, demonstrating that ischemic necrosis can occur even in actively treated patients. When clinically complicated by extensive upper extremity lymphedema, deep vein thrombosis, and malignant brachial plexopathy, it engenders a state of total pain that significantly impairs the patient's quality of life. This case demonstrates that modern palliative symptom management cannot rely solely on standard escalating opioid protocols. The integration of advanced adjuvant therapies - such as systemic lidocaine infusions, NMDA receptor antagonists, and alpha-2 adrenergic agonists - alongside individualized fungating wound care, is paramount in breaking the cycle of neuropathic suffering and maintaining patient dignity in complex advanced oncology.
